# Incremental Prognostic Value of Cystatin C-Based Estimated Glomerular Filtration Rate in Patients With Acute Coronary Syndrome

**DOI:** 10.31083/RCM39246

**Published:** 2025-10-28

**Authors:** Qiang Chen, Yike Li, Xu Chen, Haoming He, Yingying Xie, Yonghui Xu, Yue Cai, Tao Ye, Yanxiang Gao, Shiqiang Xiong, Lin Cai, Jingang Zheng

**Affiliations:** ^1^China-Japan Friendship Hospital (Institute of Clinical Medical Sciences), Chinese Academy of Medical Sciences, Peking Union Medical College, 100029 Beijing, China; ^2^Department of Cardiology, China-Japan Friendship Hospital, 100029 Beijing, China; ^3^Department of Cardiology, The Third People's Hospital of Chengdu, Affiliated Hospital of Southwest Jiaotong University, 610031 Chengdu, Sichuan, China

**Keywords:** cystatin C, estimating glomerular filtration rate, acute coronary syndrome, risk stratification

## Abstract

**Background:**

The 2021 Chronic Kidney Disease Epidemiology Collaboration (CKD-EPI) equation, which incorporates both creatinine and cystatin C, provides enhanced estimation of glomerular filtration rate (eGFR) compared to creatinine-only equations. This study aimed to explore the incremental prognostic value of eGFR estimates in patients with acute coronary syndrome (ACS).

**Method:**

This retrospective analysis evaluated 1400 ACS patients undergoing a percutaneous coronary intervention (PCI). The primary endpoint was defined as major adverse cardiovascular events (MACEs), a composite of all-cause death and nonfatal myocardial infarction (MI). The eGFR values were calculated using three equations: one based solely on serum creatinine (eGFR_cr_), another based only on cystatin C (eGFR_cys_), and a combined equation using both creatinine and cystatin C (eGFR_cys-cr_). Cox regression and the Kaplan–Meier analyses were employed to identify predictors of MACEs. The incremental prognostic value of the three eGFR equations on ACS outcomes was individually assessed.

**Results:**

Over a median follow-up of 31.03 (27.34, 35.06) months, 135 (9.6%) patients experienced MACEs, including 99 (7.1%) deaths and 41 (2.9%) MIs. Lower eGFR values correlated with higher MACEs and the risk of death. Incorporating eGFR_cys_ or eGFR_cys-cr_ into the established risk model improved the predictive accuracy for MACEs. When compared to eGFR_cr_, eGFR_cys-cr_ demonstrated greater capacity to reclassify the risk for MACEs (category-free continuous net reclassification improvement (cNRI)^>0^: 0.205 (0.011–0.397); *p* = 0.03; integrated discrimination improvement (IDI): 0.010 (0.002–0.019); *p* = 0.01), whereas eGFR_cys_ did not demonstrate a similar effect.

**Conclusion:**

The eGFR based on the 2021 CKD-EPI equation using both creatinine and cystatin C significantly improves risk prediction and reclassification in ACS patients compared with a creatinine-based equation.

## 1. Introduction

Acute coronary syndrome (ACS) is a critical and severe manifestation of coronary artery disease, associated with significant morbidity and mortality. Despite improvements in reperfusion techniques, the long-term morbidity and mortality following percutaneous coronary intervention (PCI) continue to be substantial [1]. Accurate prognostic evaluation in ACS patients is crucial for guiding clinical decision-making and optimizing therapeutic strategies. Renal impairment has been identified as a significant risk factor influencing the progression and prognosis of coronary heart disease [2, 3]. Traditional cardiovascular risk assessment tools, such as the Global Registry of Acute Coronary Events (GRACE) score, typically include estimates of renal function based on serum creatinine levels [4, 5, 6]. However, serum creatinine concentrations can be influenced by various non-renal factors, potentially limiting the accuracy in evaluating the “true” renal function [7, 8].

Cystatin C, an endogenous cysteine protease inhibitor, has been recognized as a more reliable biomarker for estimating glomerular filtration rate (eGFR) compared to serum creatinine [8, 9, 10]. Furthermore, cystatin C exhibits enhanced prognostic accuracy for cardiovascular morbidity and mortality, especially in patients with chronic kidney disease (CKD) [11], diabetes mellitus [12], atrial fibrillation [13, 14], and ACS [15, 16]. Recently, the 2021 Chronic Kidney Disease Epidemiology Collaboration (CKD-EPI) equation, which incorporates both serum creatinine and cystatin C and excludes race as a variable, offers a more accurate assessment of renal function [8]. Additionally, it enhances the prediction of heart failure [9, 17], atrial fibrillation [14], end-stage renal disease [18], and cardiovascular mortality [18] across diverse demographic populations.

While reduced renal function is an established risk factor for adverse outcomes in ACS, current prognostic models remain predominantly dependent on creatinine-based eGFR (eGFR_cr_)—a marker susceptible to fluctuations in muscle mass and inflammatory states. In contrast, cystatin C-based eGFR (eGFR_cys_) offers significant advantages as a biomarker of glomerular filtration [15, 16]. The combined equation (eGFR_cys-cr_) further refines risk stratification by integrating both biomarkers, thereby providing a more comprehensive assessment of renal function in ACS patients [19].

Despite these advancements, the incremental prognostic value of cystatin C-based eGFR using the 2021 CKD-EPI equation in ACS patients who underwent PCI has not been fully explored. This study seeks to bridge this research gap by employing the revised 2021 CKD-EPI formula that integrates both creatinine and cystatin C measurements.

## 2. Methods

### 2.1 Study Population

We enrolled a total of 1400 patients who were hospitalized at the Third People’s Hospital of Chengdu (Sichuan, China) who underwent PCI between July 2018 and December 2020. Plasma creatinine and cystatin C values were available for all enrolled patients. Exclusion criteria included: (1) history of coronary artery bypass graft (CABG); (2) Severe valvular heart disease necessitating intervention; (3) end-stage hepatic disease (Child-Pugh Class C); (4) End-stage disease of any major organ system (e.g., malignancy, respiratory failure) and an anticipated survival of <1 year; (5) mortality during hospitalization; and (6) missing >10% of essential clinical data.

### 2.2 Data Collection

Demographic information, medical history, smoking status, and specific medical details were systematically extracted from electronic health records. ACS included unstable angina, ST-segment elevation myocardial infarction (STEMI), and non-ST-segment elevation myocardial infarction (NSTEMI), as defined by corresponding guidelines [1]. Peripheral venous blood was sampled from patients after overnight fasting (>8 h) and analyzed for lipid profiles (total cholesterol, triglycerides, low density lipoprotein cholesterol (LDL-C), high density lipoprotein cholesterol (HDL-C)), metabolic parameters (fasting blood glucose, creatinine, cystatin C), cardiac troponin T (cTnT), and brain natriuretic peptide (BNP) using standardized laboratory protocols. The GRACE score was calculated [20]. The baseline Synergy between Percutaneous Coronary Intervention with TAXUS and Cardiac Surgery (SYNTAX) score (bSS) was determined using a web-based calculator: www.syntaxscore.com. Two blinded interventional cardiologists independently analyzed preprocedural angiograms, and a third cardiologist adjudicated discordant cases. All data were entered into a quality-controlled electronic database. The residual SYNTAX score (rSS) quantified residual coronary disease after PCI. For patients undergoing planned staged PCI, the rSS following the final procedure was used.

eGFR values were calculated using three different equations: one based solely on serum creatinine (eGFR_cr_), one based only on cystatin C (eGFR_cys_), and another based on a combined equation using both creatinine and cystatin C (eGFR_cys-cr_). The 2021 CKD-EPI equations [8] were used for eGFR_cr_ and eGFR_cys-cr_, while the 2012 CKD-EPI equation [21] was applied for eGFR_cys_.

### 2.3 Follow-up and Endpoints

Standardized follow-up evaluations were performed at 1, 6, and 12 months after discharge, followed by annual assessments via telephone interviews or clinical visits. Clinical outcomes were prospectively recorded by trained personnel. The primary endpoint was defined as major adverse cardiovascular events (MACEs), a composite of all-cause mortality and nonfatal myocardial infarction (MI). Secondary endpoints comprised all-cause death, cardiac death, MI, unplanned revascularization (ischemia-driven due to lesion progression or in-stent restenosis), and stroke. All events were rigorously adjudicated through a review of medical records in accordance with international diagnostic criteria.

### 2.4 Statistical Analysis

Continuous variables were summarized using mean ± standard deviation for normally distributed data or median (interquartile range) for non-normal distributions, with between-group comparisons performed using Student’s *t*-test or Mann-Whitney U test, respectively. Categorical data were expressed as number (percentage) and analyzed using χ^2^ test or Fisher’s exact test as appropriate. All statistical analyses in the present study were performed using R Programming Language 4.4.1 (R Foundation for Statistical Computing, Vienna, Austria), and MedCalc 19.1 (MedCalc Software Ltd, Ostend, Belgium). All tests were two-sided with significance at *p* < 0.05.

Participants were categorized into four groups based on different eGFR levels as determined by the eGF_cr_, eGFR_cys_, and eGFR_cys-cr_ equations, respectively. For each equation, the grouping was as follows: T1: eGFR <30 mL/min/1.73 m^2^; T2: 30 ≤ eGFR < 60 mL/min/1.73 m^2^; T3: 60 ≤ eGFR < 90 mL/min/1.73 m^2^; T4: eGFR ≥90 mL/min/1.73 m^2^. To better represent the relationship between declining kidney function and adverse cardiovascular outcomes, eGFR values were transformed by taking the negative and then dividing by 10 before being included in Cox regression analyses as continuous variables. The cumulative incidence of the adverse cardiovascular events was evaluated using the Kaplan-Meier method and compared across groups with the log-rank test. The hazard ratio (HR) and 95% confidence interval (CI) for developing the MACEs were estimated using the univariate and multivariate Cox proportional hazards regression model. The predictive capacities of different variables evaluating renal function were assessed by calculating the area under the Receiver Operating Characteristic (ROC) curve (AUC) and compared using DeLong’s test.

Likelihood ratio tests (χ^2^) evaluated improvement in model fit after incorporating each eGFR measure (eGFR_cr_, eGFR_cys_, eGFR_cys-cr_). The baseline model covariates were determined through a dual evidence-driven approach: (1) initial screening of variables with univariate association (*p* < 0.05) with MACEs, followed by (2) strict retention of clinically established prognosticators. For both nested and non-nested model comparisons, we computed: Corrected Akaike’s Information Criterion (AICc), Delta-AICc (ΔAICc), and Bayesian Information Criterion (BIC). Bootstrap validation with 1000 resamples assessed model stability. The relatively corrected C-index quantified the model’s ability to differentiate risk outcomes. Brier scores and calibration curves evaluated the agreement between predicted probabilities and observed event rates. We calculated category-free continuous net reclassification improvement (cNRI^>0^) and integrated discrimination improvement (IDI) to measure the incremental predictive value of each eGFR equation. Decision curve analysis (DCA) was employed to assess the clinical utility.

## 3. Results

### 3.1 Baseline Characteristics Stratified by the Occurrence of MACEs

The final analysis included 1400 eligible patients, with a mean age of 67.09 ± 11.28 years. Over a median follow-up of 31.03 (IQR 27.34–35.06) months, 135 (9.6%) MACEs occurred, comprising 99 (7.1%) all-cause deaths and 41 (2.9%) MIs. Baseline characteristics are detailed in Table 1. Participants who experienced MACEs were generally older and had higher levels of heart rate, cTnT, BNP, serum creatinine, cystatin C, as well as higher GRACE scores and bSS. They also exhibited lower left ventricular ejection fraction (LVEF), body mass index (BMI), and eGFR values calculated using eGFR_cr_, eGFR_cys_, eGFR_cys-cr_, equations, respectively. Additionally, these individuals had a higher incidence of chronic obstructive pulmonary disease (COPD), acute myocardial infarction (AMI), and diabetes mellitus (DM), with greater usage of insulin and diuretics upon discharge, compared to those without MACEs. Among the population experiencing MACEs, the distribution of those with eGFR <60 mL/min/1.73 m^2^, as determined by different equations, was as follows: 44 (32.6%) for eGFR_cr_, 97 (71.9%) for eGFR_cys_, and 62 (45.9%) for eGFR_cys-cr_. Baseline characteristics stratified by the categories of GFR_cys-cr_ are detailed in **Supplementary Table 1**.

**Table 1.** **Baseline characteristics stratified by the occurrence of MACEs**.

Variable		No MACEs	MACEs	*p* value
		(n = 1265)	(n = 135)	
Age, years		66.35 ± 11.18	73.98 ± 9.79	<0.001
Female, n (%)		356 (28.1)	49 (36.3)	0.047
sBMI, kg/m^2^		24.45 ± 2.97	23.85 ± 2.97	0.024
Smoking, n (%)		680 (53.8)	62 (45.9)	0.083
Previous PCI, n (%)		105 (8.3)	16 (11.9)	0.163
COPD, n (%)		34 (2.7)	10 (7.4)	0.003
Hypertension, n (%)		852 (67.4)	99 (73.3)	0.157
Diabetes mellitus, n (%)		489 (38.7)	69 (51.1)	0.005
Previous stroke, n (%)		60 (4.8)	6 (4.4)	0.870
SBP, mmHg		133.12 ± 20.84	133.39 ± 22.60	0.889
Heart rate, bpm		76.77 ± 13.91	80.37 ± 17.47	0.005
Laboratory Measurements				
	Creatinine, mg/dL	0.86 (0.74, 1.02)	0.97 (0.76, 1.37)	<0.001
	Cystatin C, mg/L	1.13 (0.96, 1.37)	1.37 (1.12, 2.11)	<0.001
	eGFR_cr_, mL/min/1.73 m^2^	91.43 (74.68, 99.89)	76.05 (49.63, 91.75)	<0.001
	eGFR_cr_ <60 mL/min/1.73 m^2^	156 (12.3)	44 (32.6)	<0.001
	eGFR_cys_, mL/min/1.73 m^2^	63.37 (47.63, 79.52)	47.02 (26.73, 62.69)	<0.001
	eGFR_cys_ <60 mL/min/1.73 m^2^	565 (44.7)	97 (71.9)	<0.001
	eGFR_cys-cr_, mL/min/1.73 m^2^	77.50 (61.11, 92.38)	62.19 (35.77, 76.14)	<0.001
	eGFR_cys-cr_ <60 mL/min/1.73 m^2^	305 (24.1)	62 (45.9)	<0.001
	cTnT, pg/mL	51.00 (11.60, 1211.90)	194.40 (25.40, 1507.00)	<0.001
	BNP, pg/mL	117.90 (40.95, 328.49)	328.49 (93.30, 999.00)	<0.001
	FBG, mmol/L	6.93 ± 3.23	7.22 ± 3.01	0.312
	TG, mmol/L	1.87 ± 1.38	1.66 ± 0.93	0.089
	TC, mmol/L	4.48 ± 1.26	4.45 ± 1.22	0.789
	HDL-C, mmol/L	1.16 ± 0.30	1.15 ± 0.29	0.855
	LDL-C, mmol/L	2.74 ± 0.92	2.73 ± 0.89	0.872
	LVEF	55.38 ± 8.33	51.36 ± 11.14	<0.001
	AMI, n (%)	620 (49.0)	82 (60.7)	0.010
Diagnosis, n (%)				0.009
	Unstable angina	645 (51.0)	53 (39.3)	
	NSTEMI	262 (20.7)	42 (31.1)	
	STEMI	358 (28.3)	40 (29.6)	
	bSS	13.00 (8.00, 19.50)	19.00 (12.00, 27.50)	<0.001
	rSS	3.00 (0.00, 7.00)	5.00 (1.00, 10.50)	<0.001
	GRACE score	103.30 ± 29.72	130.36 ± 31.59	<0.001
Discharge medications				
	Statins, n (%)	1240 (98.0)	132 (97.8)	0.846
	β-blockers, n (%)	878 (69.4)	93 (68.9)	0.901
	ACEI/ARB, n (%)	569 (45.0)	69 (51.1)	0.174
	Diuretics, n (%)	191 (15.1)	51 (37.8)	<0.001
	Insulin, n (%)	123 (9.7)	24 (17.8)	0.004

**Abbreviations**: GRACE score, Global Registry of Acute Coronary Events score; BMI, body mass index; PCI, percutaneous coronary intervention; COPD, chronic obstructive pulmonary disease; SBP, systolic blood pressure; FBG, fasting blood glucose; TG, triglyceride; TC, total cholesterol; HDL-C, high density lipoprotein cholesterol; LDL-C, low density lipoprotein cholesterol; cTnT, cardiac troponin T; BNP, brain natriuretic peptide; AMI, acute myocardial infarction; UA, unstable angina; STEMI, ST-segment elevation myocardial infarction; NSTEMI, non-ST-segment elevation myocardial infarction; ACEI/ARB, angiotensin converting enzyme inhibitor/angiotensin receptor blocker; bSS, baseline SYNTAX score; LVEF, left ventricular ejection fraction; eGFR, estimated glomerular filtration rate; eGFR_cys-cr_, eGFR calculated using a combined creatinine-cystatin C method; eGFR_cys_, eGFR calculated using cystatin C only; eGFR_cr_, eGFR calculated using creatinine only; MACEs, major adverse cardiovascular events.

### 3.2 The Predictive Value of Different eGFR Equations for Adverse Cardiovascular Events

**Fig. 1.**
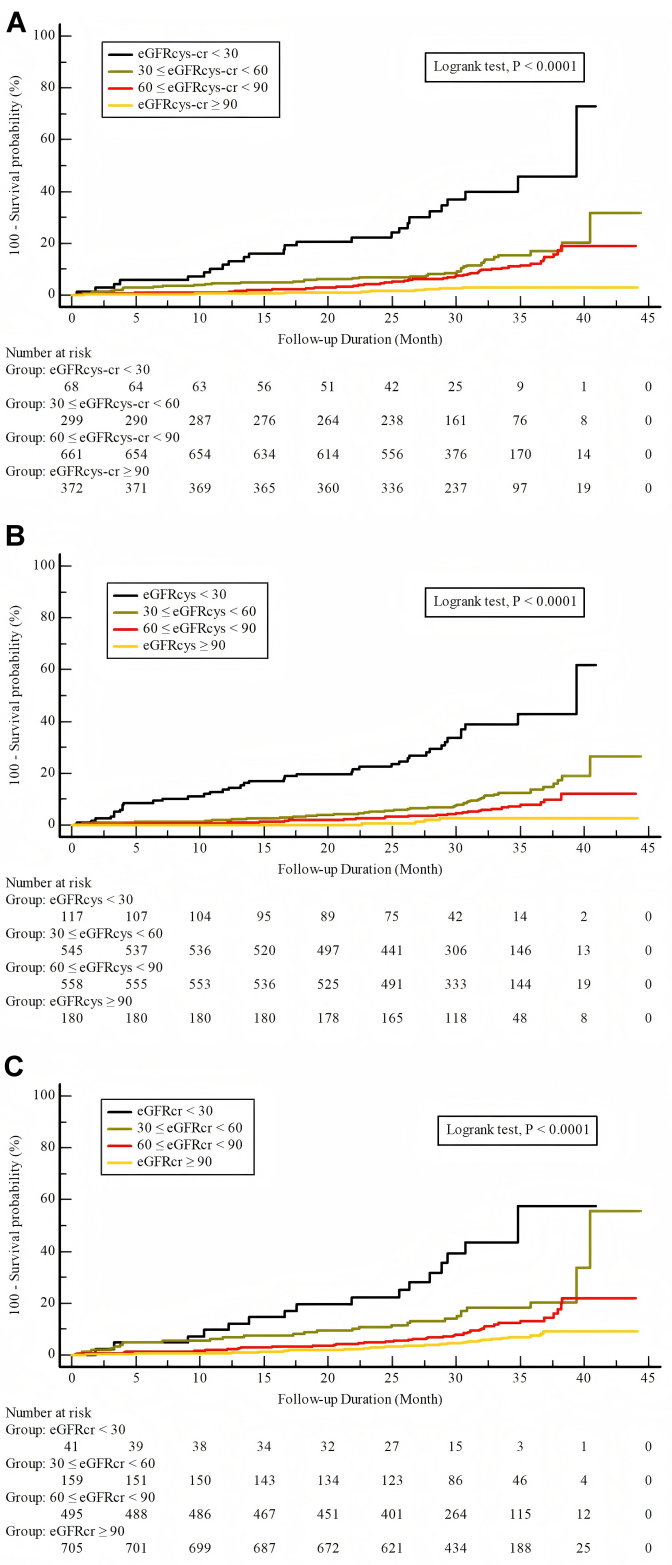
**Incidence of MACEs stratified by eGFR categories**. Incidence of MACEs stratified by eGFR categories as estimated by three different equations. Each panel represents results from a different eGFR estimation method. (A) Combined creatinine-cystatin C equation (eGFR_cys-cr_). (B) Cystatin C-based equation (eGFR_cys_). (C) Creatinine-based equation (eGFR_cr_).

We observed a significant and progressive increase in the incidence of MACEs, all cause death and cardiac death in groups with reduced eGFR values (*p* < 0.001, **Supplementary Table 2**). The group with the lowest eGFR category (T1) exhibited the highest incidence of MACEs. However, no significant differences were observed in the rates of MI, unplanned revascularization across the groups (*p* > 0.050). Kaplan-Meier survival analysis demonstrated the differences in the incidence of MACEs (Fig. 1A–C) across varying eGFR categories, determined using eGFR_cr_, eGFR_cys_, and the eGFR_cys-cr_ equations (*p* < 0.001).

**Fig. 2.**
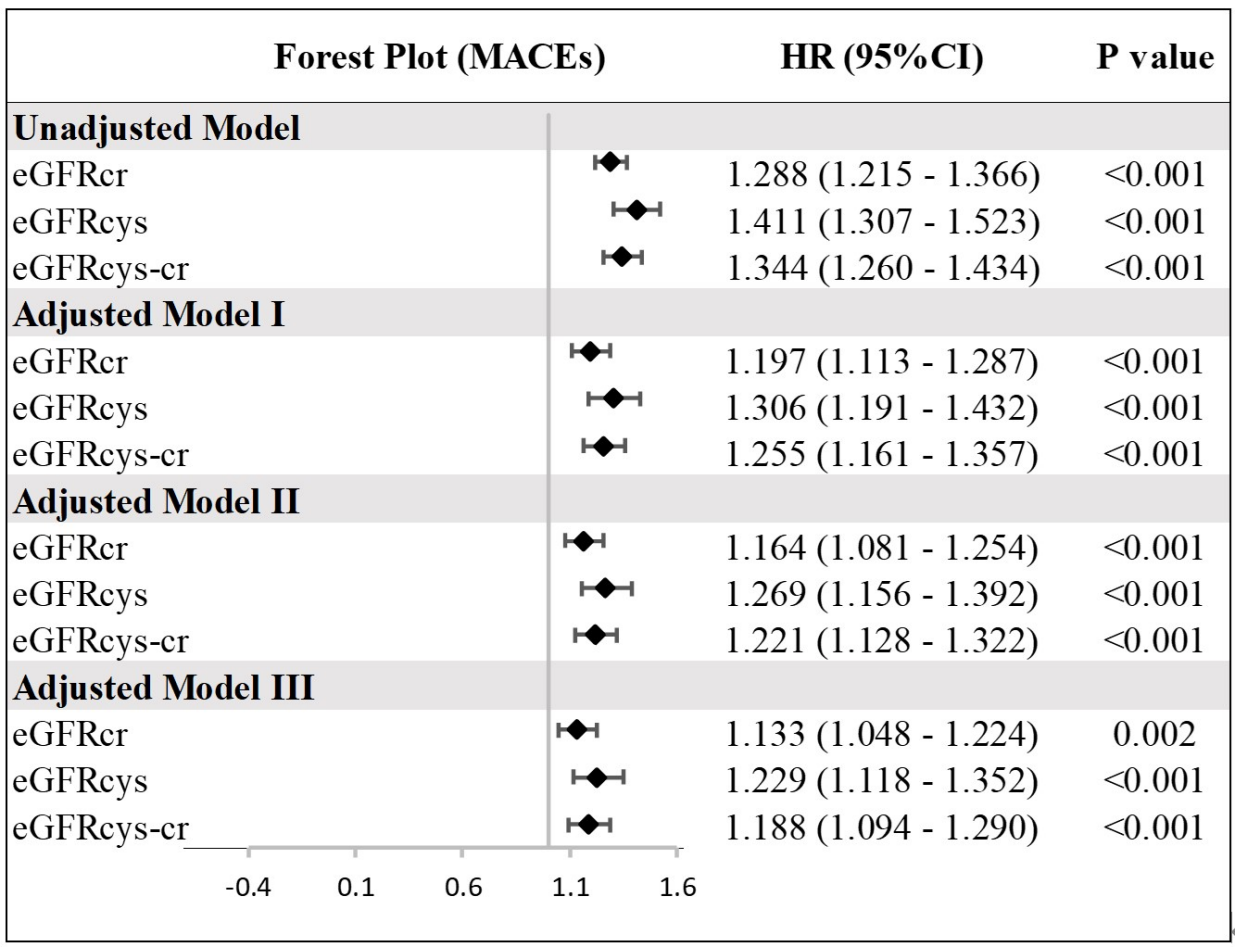
**Multivariate analysis of MACEs by different eGFR calculated using three equations**. Hazard ratio (HR) indicates an increased risk for each 10-unit decrease in eGFR. Model I was adjusted for age, BMI, sex, HTN, DM, smoking; Model II was adjusted for Model I plus Heart rate, AMI, LVEF; Model III was adjusted for Model II plus Diuretics, Insulin, bSS. HTN, hypertension; DM, diabetes mellitus.

The univariate Cox regression analysis consistently demonstrated that lower eGFR values, as determined by the eGFR_cr_, eGFR_cys_, and eGFR_cys-cr_ equations, are associated with an increased risk of MACEs (**Supplementary Table 3**). These associations remained significant even after adjusting for confounders in different multivariate Cox regression models as illustrated in the forest plot (Fig. 2). This underscores the critical role of kidney function as a predictor of adverse cardiovascular prognosis in ACS.

**Fig. 3.**
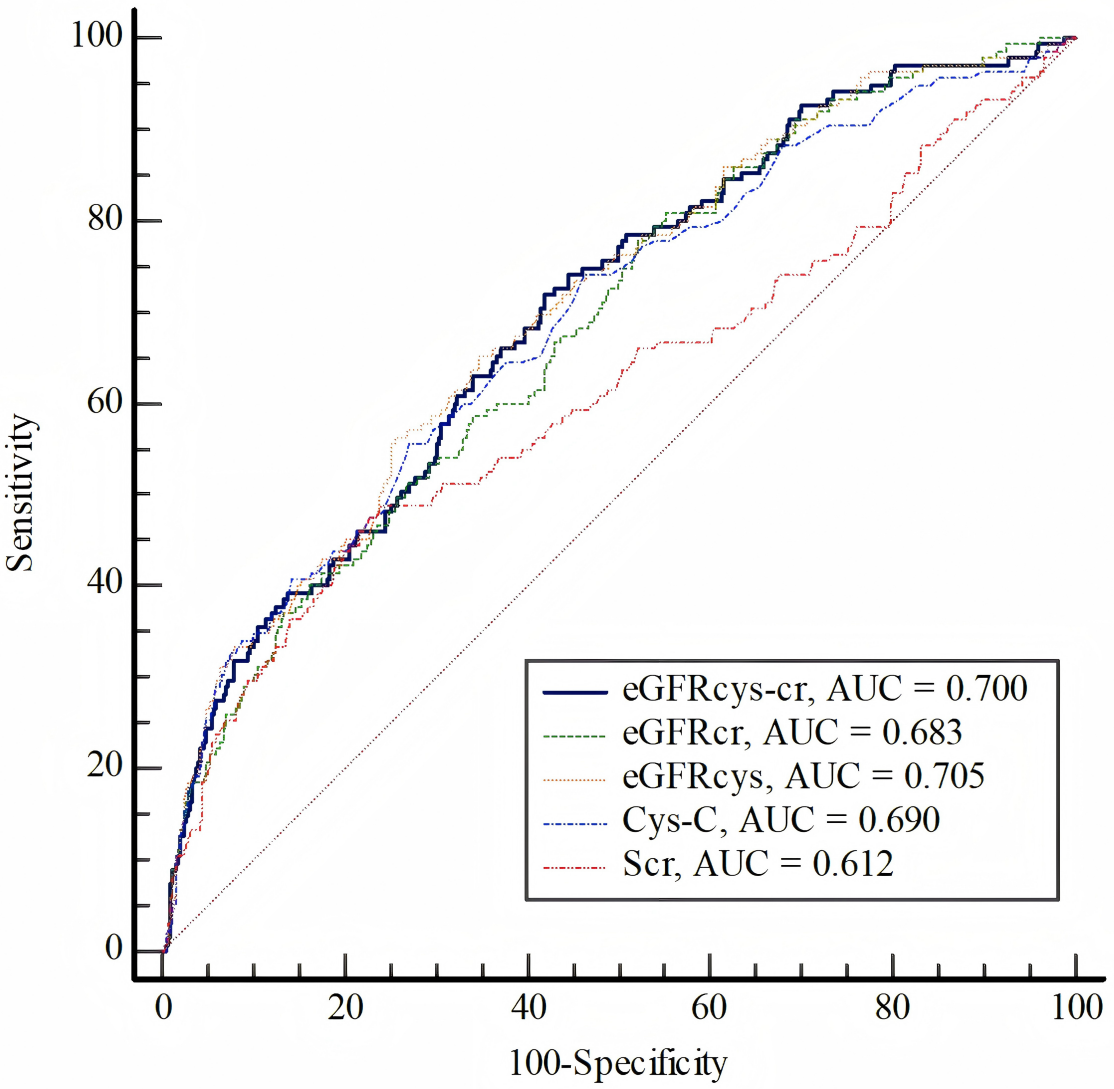
**Receiver Operating Characteristic (ROC) analysis of the eGFR_cys-cr_, eGFR_𝐜𝐲𝐬_, eGFR_𝐜𝐫_, Creatinine and Cystatin C to predict MACEs**.

The ROC analysis indicated (Fig. 3, **Supplementary Tables 4,5**) that using eGFR as determined by any of these equations (eGFR_cys-cr_, eGFR_cys_, eGFR_cr_) provides superior predictive performance for MACEs compared to using serum creatinine alone (*p* < 0.001). Notably, both eGFR_cys-cr_ [AUC = 0.700, 95% CI (0.653–0.746), *p* < 0.001] and eGFR_cys_ [AUC = 0.705, 95% CI (0.658–0.752), *p* < 0.001] had higher AUCs compared to eGFR_cr_ [AUC = 0.683, 95% CI (0.637–0.730), *p* < 0.001] for MACEs, respectively.

### 3.3 Performance of Prediction Models Incorporating Different eGFR Measures for MACEs

The performance of integrating various eGFR measures into the baseline risk model for ACS undergoing PCI was analyzed. Likelihood ratio tests indicated significant enhancements in the prediction of MACEs when these eGFR metrics were added as continuous variables to the baseline risk model (Table 2). Models incorporating eGFR_cys_ or eGFR_cys-cr_ demonstrated lower corrected AIC and BIC values compared to those incorporating eGFR_cr_, indicating an improved model fit (Table 2).

**Table 2.** **Evaluation of predictive models for MACEs with AIC, BIC and likelihood ratio criteria**.

	Akaike’s information criteria		BIC	Likelihood ratio test		
	AICc	δAICc		χ ^2^	df	*p* value
Baseline risk model	1748.82	16.20	1783.45	ref	ref	ref
Baseline risk model + eGFR_cr_	1741.67	9.05	1779.18	9.18	1.00	<0.01
Baseline risk model + eGFR_cys_	1732.62	0.00	1770.13	18.23	1.00	<0.01
Baseline risk model + eGFR_cys-cr_	1734.76	2.14	1772.26	16.09	1.00	<0.01

Baseline risk model was adjusted for age, BMI, sex, HTN, DM, smoking, heart rate, AMI, LVEF, Diuretics, Insulin, bSS. HTN, hypertension; DM, diabetes mellitus; eGFR, estimated glomerular filtration rate; eGFR_cys-cr_, eGFR calculated using a combined creatinine-cystatin C method; eGFR_cys_, eGFR calculated using cystatin C only; eGFR_cr_, eGFR calculated using creatinine only; AICc, corrected Akaike’s information criterion; δAICc, delta-AICc; BIC, Bayesian information criterion.

The performance of these models, as estimated through internal bootstrap validation for predicting MACEs, is presented in **Supplementary Table 6**. The baseline risk model exhibited moderate discrimination with C-indices of 0.770. The inclusion of eGFR_cr_, eGFR_cys_, or eGFR_cys-cr_ into the model significantly enhanced the C-index, with the highest improvement observed in models integrating eGFR_cys_ (C-index: 0.780 for the baseline risk model). Additionally, the Brier scores for the baseline risk model were 0.144. Models including eGFR_cys_ or eGFR_cys-cr_ displayed lower Brier scores (0.154), indicating superior calibration. Calibration plots for MACEs demonstrated strong agreement between observed outcomes and predicted probabilities (**Supplementary Fig. 1**).

### 3.4 Incremental Predictive Value of Cystatin C-based eGFR for MACEs

**Fig. 4.**
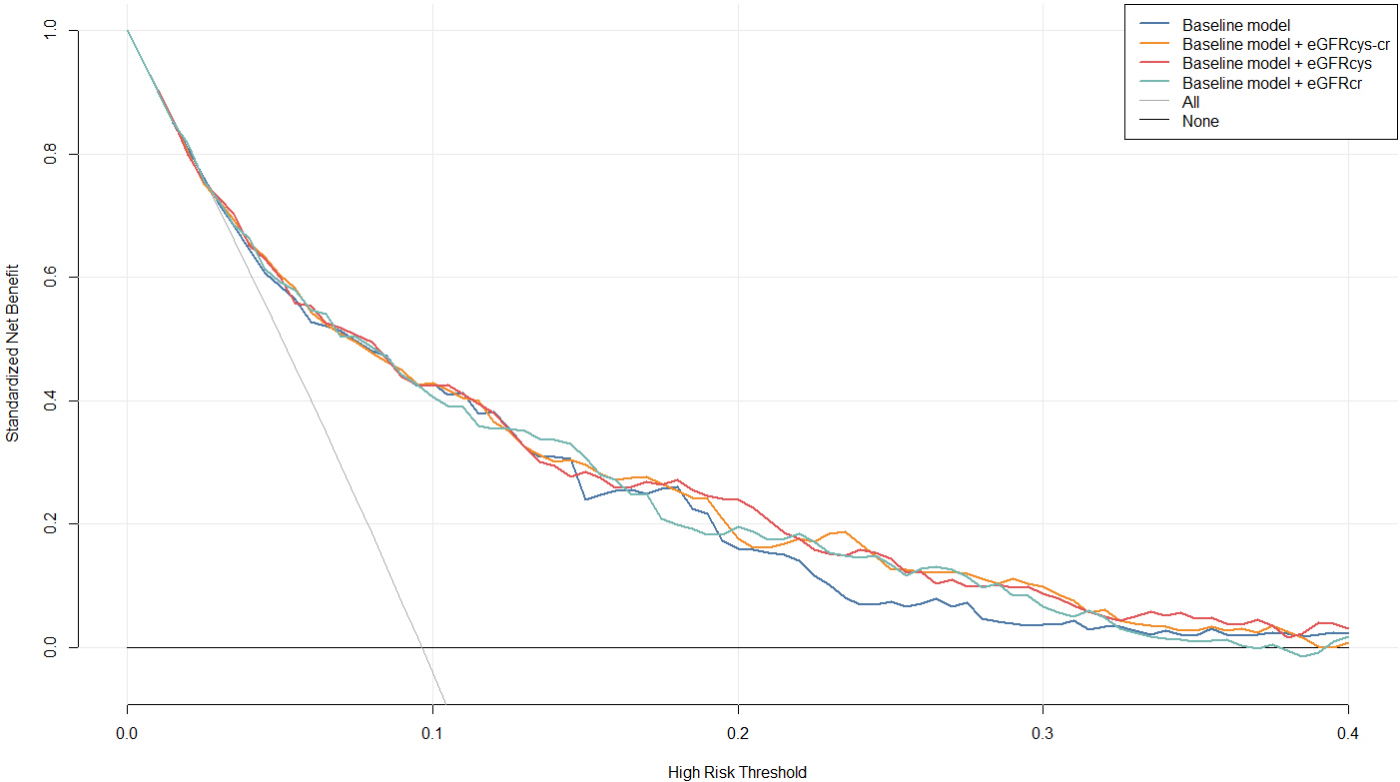
**Decision Curve Analysis (DCA)**. Net benefits for predicting MACEs using the baseline risk model and models incorporating eGFR_cr_, eGFR_cys_, and eGFR_cys-cr_. Baseline model was adjusted for age, BMI, sex, HTN, DM, smoking, Heart rate, AMI, LVEF, Diuretics, Insulin, bSS. HTN, hypertension; DM, diabetes mellitus.

The addition of eGFR_cys_ or eGFR_cys-cr_ to the baseline risk model resulted in significant improvements in risk reclassification for MACEs (Table 3), as assessed by cNRI^>0^ (all *p* < 0.05) and IDI (all *p* < 0.05). Additionally, eGFR_cys_ provided the highest incremental predictive value in improving the baseline risk model [cNRI^>0^: 0.187 (0.034–0.321), *p* = 0.02; IDI: 0.015 (0.003–0.045), *p* = 0.02] for MACEs. Conversely, incorporating eGFR_cr_ did not significantly enhance net reclassification [cNRI^>0^: 0.118 (–0.099–0.255), *p* = 0.209] or integrated discrimination [IDI: 0.007 (–0.001–0.028), *p* = 0.169]. When compared to eGFR_cr_, eGFR_cys-cr_ alone significantly improved the reclassification of MACEs [cNRI^>0^: 0.205 (0.011–0.397), *p* = 0.03; IDI: 0.010 (0.002–0.019), *p* = 0.01], whereas eGFR_cys_ alone did not demonstrate a similar effect. Models incorporating eGFR_cys_ and eGFR_cys-cr_ consistently exhibited higher net benefits across most high-risk thresholds (Fig. 4).

**Table 3.** **The incremental predictive value of various eGFR estimation equation**.

	cNRI^>0^ (95% CI)	*p*	IDI (95% CI)	*p*
eGFR_cr_	Ref.	Ref.	Ref.	Ref.
eGFR_cys_	0.154 (–0.044, 0.349)	0.119	0.012 (–0.001, 0.027)	0.080
eGFR_cys-cr_	0.205 (0.011, 0.397)	0.030	0.010 (0.002, 0.019)	0.010
Baseline risk model	Ref.	Ref.	Ref.	Ref.
Baseline risk model + eGFR_cr_	0.118 (–0.099, 0.255)	0.209	0.007 (–0.001, 0.028)	0.169
Baseline risk model + eGFR_cys_	0.187 (0.034, 0.321)	0.021	0.015 (0.003, 0.045)	0.020
Baseline risk model + eGFR_cys-cr_	0.124 (0.018, 0.290)	0.032	0.013 (0.001, 0.046)	0.039

Baseline risk model was adjusted for age, sex, BMI, HTN, DM, smoking, Heart rate, AMI, LVEF, Diuretics, Insulin, bSS. HTN, hypertension; DM, diabetes mellitus.

## 4. Discussion

Our findings demonstrate that incorporating cystatin C-based eGFR, particularly eGFR_cys-cr_ using the CKD-EPI 2021 equations, significantly enhances the prediction of MACEs in this population compared to traditional creatinine-based measures alone. This suggests creatinine-cystatin C-based renal assessment may refine therapeutic and monitoring protocols in ACS patients after PCI.

Impaired renal function results in alterations in calcium and phosphorus metabolism, endothelial dysfunction, and increases oxidative stress and lipid metabolism disorders [3, 22, 23]. These factors independently contribute to the onset and poor prognosis of cardiovascular diseases, significantly elevating the risk of adverse cardiovascular events in patients with ACS [3]. Traditionally, serum creatinine levels and creatinine-based estimation formulas, such as the Modification of Diet in Renal Disease (MDRD) [24] or CKD-EPI equations [21, 25], are commonly used to calculate the eGFR. In recent years, eGFR estimation based on cystatin C, as well as combined creatinine and cystatin C estimation, has been shown to provide more accurate assessments of kidney function in certain diseases [8]. Higher cystatin C levels were associated with 2.53-fold mortality risk and 3.24-fold MACE risk in ACS patients [26]. Prior studies demonstrated that cystatin C-containing CKD-EPI equations (2012) outperform creatinine-based eGFR for ACS risk stratification [19]. In our study, lower eGFR values, as determined by the eGFR_cr_, eGFR_cys_, and eGFR_cys-cr_ equations, were associated with an increased risk of MACEs, resulting in better predictive values than using serum creatinine alone.

We found that incorporating cystatin C-based eGFR (eGFR_cys_ or eGFR_cys-cr_) improves the predictive capability of the established risk models for MACEs. However, eGFR_cr_ alone did not enhance the predictive ability for adverse cardiovascular events. This is generally consistent with previous research findings. The Stabilization of Plaques Using Darapladib-Thrombolysis in Myocardial Infarction (SOLID-TIMI) 52 trial showed that cystatin C improved the prediction of cardiovascular disease/heart failure (CVD/HF) hospitalization when added to a non-eGFR adjusted model in ACS [15]. Inês Almeida *et al*. [19] found that eGFR_cys_, calculated using the CKD-EPI 2012 equation, offers a novel and superior method for assessing mortality risk in patients admitted for ACS. This method outperforms the MDRD-4 equation and enhances the predictive value of the GRACE score [19]. The superior performance of eGFR_cys_ and eGFR_cys-cr_ in predicting cardiovascular outcomes can be attributed to several factors related to their underlying pathophysiology. Cystatin C is less influenced by muscle mass, inflammation, gender, ethnicity, and diet compared to serum creatinine, offering a more consistent reflection of the glomerular filtration rate across diverse patient populations, serving as a more sensitive and specific biomarker for the early detection of impaired kidney function [7, 8, 27]. Consequently, this leads to more accurate risk stratification, which is particularly critical in the ACS population where comorbidities such as frailty and malnutrition can alter creatinine-based estimates.

Furthermore, our study revealed that, despite the AUC value of eGFR_cys_ being greater than that of eGFR_cys-cr_, the latter remarkably improved the reclassification of MACEs compared to eGFR_cr_. In contrast, eGFR_cys_ did not demonstrate a similar benefit, which is consistent with previous research findings. Although cystatin C is more sensitive than serum creatinine for detecting early impairment of kidney function, it is also influenced by factors such as obesity, smoking, inflammation, thyroid activity, and glucocorticoid levels, which can result in potential overestimation of kidney damage [8, 28]. Numerous studies have shown that the accuracy of eGFR using both cystatin C and creatinine, which reduces the measurement errors associated with both filtration markers, surpasses that of estimates using either creatinine or cystatin C alone [8, 21, 29]. Besides, our study demonstrates that among individuals who have experienced MACEs, the proportion of patients identified with an eGFR of less than 60 mL/min/1.73 m^2^ by eGFR_cys_ [97 (71.9%)] is significantly higher than those identified using eGFR_cys-cr_ [62 (45.9%)] or eGFR_cr_ [44 (32.6%)]. Statistically, eGFR_cys_ may detect a greater number of patients with an adverse prognosis in various patient populations. However, it also tends to classify more borderline cases as high-risk, which may increase the risk of misclassification. Consequently, from the perspective of precision medicine and individualized risk prediction, the eGFR_cys-cr_ equation may provide superior reclassification capabilities in clinical practice, thus enhancing its clinical utility.

The 2024 Kidney Disease: Improving Global Outcomes (KDIGO) guidelines for the management and evaluation of CKD recommend using an eGFR that combines cystatin C and creatinine to stage kidney function [7]. Additionally, they advise that cardiovascular risk prediction models for CKD patients should incorporate both eGFR and proteinuria [7]. In 2023, the American Heart Association (AHA) issued a Presidential Advisory on cardiovascular-renal-metabolic syndrome (CKM), highlighting the relationship of cardiovascular disease, CKD, and metabolic disorders [30]. The advisory emphasized the necessity of integrating kidney function into cardiovascular risk assessments. This underscores the need for more precise and individualized evaluation of kidney function. Moreover, traditional prognostic models for ACS patients, such as the GRACE score [20], often overlook kidney function or assess it solely based on serum creatinine levels, which is inadequate for precise, personalized management.

Thus, incorporating cystatin C into existing prognostic models has significant clinical implications, particularly for ACS patients. First, it enhances risk stratification by more accurately identifying high-risk individuals, enabling clinicians to allocate more intensive monitoring and targeted therapeutic interventions [7, 8]. This is crucial for ACS patients, as refined stratification allows for better management of conditions affecting muscle mass or in those with advanced age, which are common challenges in creatinine-based assessments. Moreover, the updated CKD-EPI 2021 equations, which incorporate both cystatin C and creatinine, ensure equitable prognostication across racial and ethnic populations by eliminating race as a variable. This equitable approach is essential in providing personalized care that meets the diverse needs of ACS patients.

Despite the robustness of our study, several limitations must be acknowledged. First, although we thoroughly adjusted for clinical and biochemical variables, residual confounding—notably potential interference from contrast agents on renal function biomarkers—persists due to the inherent limitations of our retrospective observational design. Second, the generalization of our findings may be constrained by the study’s single-center design based on a Chinese population. Future multicenter studies that include a more diverse cohort would be necessary to validate these findings across different populations. Furthermore, our current approach evaluates the impact of the eGFR measured at a single point during hospitalization on long-term prognosis. However, fluctuations in kidney function throughout the hospital stay may introduce bias. Future research should consider assessing kidney function immediately before discharge to enhance the predictive value for long-term outcomes and minimize potential bias. Additionally, while eGFR_cys_ and eGFR_cys-cr_ provide improved prognostic value, the integration of even more specific biomarkers associated with inflammation, endothelial dysfunction, and cardiovascular stress could further enhance prediction models. Emerging biomarkers in cardiovascular and renal pathology, such as genomic and metabolic markers [31], could be explored in conjunction with cystatin C to develop more comprehensive risk stratification tools.

## 5. Conclusion

In summary, our study highlights the significant incremental prognostic value of cystatin C-based eGFR equation, particularly the CKD-EPI 2021 equation that incorporates both cystatin C and creatinine, in ACS patients when compared to creatinine-based eGFR equation. This approach will aid in risk stratification and the development of precise, individualized secondary prevention strategies for ACS patients.

## Availability of Data and Materials

The datasets used and analyzed in the study are available from the corresponding author upon reasonable request.

## Supplementary Material

Supplementary material associated with this article can be found, in the online version, at https://doi.org/10.31083/RCM39246.
