# Supplementary material for: Incremental Prognostic Value of Cystatin C-Based Estimated Glomerular Filtration Rate in Patients With Acute Coronary Syndrome
Source: Rev Cardiovasc Med. 2025 Oct 28;26(10):39246. doi: 10.31083/RCM39246 (PMC12593831; doi:10.31083/RCM39246)

**Supplementary Table 1. Baseline characteristics stratified by the categories of GFR_cys-cr_**

| Variable | T1 (n=68) | T2 (n=299) | T3 (n=661) | T4 (n=372) | P value |
| --- | --- | --- | --- | --- | --- |
| Age, years | 73.13 ± 9.97 | 73.99 ± 9.14 | 68.57 ± 9.26 | 57.81 ± 10.34 | <.001 |
| Female, n (%) | 29 (42.65) | 118 (39.46) | 170 (25.72) | 88 (23.66) | <.001 |
| BMI, kg/m^2^ | 24.55 ± 2.70 | 24.32 ± 3.13 | 24.33 ± 2.97 | 24.55 ± 2.88 | 0.626 |
| Smoking, n (%) | 28 (41.18) | 135 (45.15) | 352 (53.25) | 227 (61.02) | <.001 |
| Previous PCI, n (%) | 7 (10.29) | 36 (12.04) | 51 (7.72) | 27 (7.26) | 0.101 |
| COPD, n (%) | 4 (5.88) | 18 (6.02) | 20 (3.03) | 2 (0.54) | <.001 |
| Hypertension, n (%) | 62 (91.18) | 239 (79.93) | 467 (70.65) | 183 (49.19) | <.001 |
| Diabetes mellitus, n (%) | 40 (58.82) | 148 (49.50) | 224 (33.89) | 146 (39.25) | <.001 |
| Previous Stroke, n (%) | 6 (8.82) | 24 (8.03) | 29 (4.39) | 7 (1.88) | <.001 |
| SBP, mmHg | 135.91 ± 27.55 | 133.35 ± 22.16 | 134.30 ± 20.93 | 130.43 ± 18.48 | 0.024 |
| Heart rate, bpm | 80.71 ± 18.99 | 77.27 ± 15.54 | 76.57 ± 13.46 | 77.29 ± 13.74 | 0.150 |
| Laboratory Measurements |  |  |  |  |  |
| Creatinine, mg/dl | 2.50 (1.67,5.33) | 1.14 (1.00,1.33) | 0.86 (0.76,0.97) | 0.73 (0.62,0.82) | <.001 |
| Cystatin C, mg/L | 3.37 (2.66,6.36) | 1.60 (1.46,1.83) | 1.14 (1.06,1.24) | 0.89 (0.82,0.95) | <.001 |
| eGFR_cr_, mL/min/1.73 m^2^ | 24.64 (9.81,34.99) | 61.22 (51.84,70.48) | 90.15 (81.53,95.96) | 103.84 (99.18,109.53) | <.001 |
| eGFR_cys_, mL/min/1.73 m^2^ | 14.25 (6.15,19.87) | 38.20 (32.30,43.01) | 61.91 (55.09,69.36) | 89.51 (82.08,99.76) | <.001 |
| eGFR_cys-cr_, mL/min/1.73 m^2^ | 19.01 (9.32,25.75) | 48.75 (41.53,55.96) | 75.62 (69.33,82.70) | 100.28 (94.49,108.00) | <.001 |
| cTnT, pg/ml | 200.55 (58.17,999.95) | 56.40 (18.30,1211.90) | 31.60 (11.00,1211.90) | 119.70 (9.05,1211.90) | <.001 |
| BNP, pg/ml | 477.50 (151.15,1328.25) | 241.20 (78.10,610.85) | 108.30 (37.90,328.49) | 88.65 (33.33,247.60) | <.001 |
| FBG, mmol/L | 7.97 ± 4.18 | 7.39 ± 3.86 | 6.46 ± 2.48 | 7.31 ± 3.47 | <.001 |
| TG, mmol/L | 1.93 ± 1.04 | 1.73 ± 1.12 | 1.74 ± 1.13 | 2.10 ± 1.81 | <.001 |
| TC, mmol/L | 4.35 ± 1.20 | 4.30 ± 1.26 | 4.47 ± 1.24 | 4.66 ± 1.29 | 0.003 |
| HDL-C, mmol/L | 1.09 ± 0.29 | 1.15 ± 0.32 | 1.18 ± 0.29 | 1.15 ± 0.29 | 0.043 |
| LDL-C, mmol/L | 2.63 ± 0.88 | 2.60 ± 0.88 | 2.73 ± 0.92 | 2.88 ± 0.95 | <.001 |
| LVEF | 50.44 ± 9.92 | 53.42 ± 10.25 | 56.02 ± 8.31 | 55.27 ± 7.34 | <.001 |
| AMI, n (%) | 48 (70.59) | 153 (51.17) | 294 (44.48) | 207 (55.65) | <.001 |
| Diagnosis, n (%) |  |  |  |  | <.001 |
| Unstable angina | 20 (29.41) | 146 (48.83) | 367 (55.52) | 165 (44.35) |  |
| NSTEMI | 35 (51.47) | 74 (24.75) | 135 (20.42) | 60 (16.13) |  |
| STEMI | 13 (19.12) | 79 (26.42) | 159 (24.05) | 147 (39.52) |  |
| bSS | 19.00 (12.75,26.12) | 15.00 (9.00,22.50) | 13.00 (8.00,20.00) | 12.00 (7.00,18.00) | <.001 |
| rSS | 6.50 (2.00,11.00) | 4.00 (0.00,8.00) | 3.00 (0.00,8.00) | 2.00 (0.00,6.00) | <.001 |
| GRACE score | 169.24 ± 41.18 | 152.57 ± 35.73 | 134.84 ± 36.07 | 123.17 ± 37.42 | <.001 |
| Discharge medications |  |  |  |  |  |
| Statins, n (%) | 65 (95.59) | 291 (97.32) | 653 (98.79) | 363 (97.58) | 0.161 |
| β-blockers, n (%) | 51 (75.00) | 199 (66.56) | 456 (68.99) | 265 (71.24) | 0.426 |
| ACEI/ARB, n (%) | 22 (32.35) | 161 (53.85) | 317 (47.96) | 138 (37.10) | ＜.001 |
| Diuretics, n (%) | 34 (50.00) | 88 (29.43) | 88 (13.31) | 32 (8.60) | ＜.001 |
| Insulin, n (%) | 20 (29.41) | 46 (15.38) | 48 (7.26) | 33 (8.87) | <.001 |

**Abbreviations:** GRACE score, Global Registry of Acute Coronary Events score; BMI, body mass index; PCI, percutaneous coronary intervention; COPD, chronic obstructive pulmonary disease; SBP, systolic blood pressure; FBG, fasting blood glucose; TG, triglyceride; TC, total cholesterol; HDL-C, high density lipoprotein cholesterol; LDL-C, low density lipoprotein cholesterol; cTnT, cardiac troponin T; BNP, brain natriuretic peptide; AMI, acute myocardial infarction; UA, unstable angina; STEMI, ST-segment elevation myocardial infarction; NSTEMI, non-ST-segment elevation myocardial infarction; ACEI/ARB, angiotensin converting enzyme inhibitor/angiotensin receptor blocker; bSS, baseline SYNTAX score; LVEF, left ventricular ejection fraction;

T1: eGFR_cys-cr_ <30 mL/min/1.73 m²; T2: 30 ≤ eGFR_cys-cr_ < 60 mL/min/1.73 m²; T3: 60 ≤ eGFR_cys-cr_ < 90 mL/min/1.73 m²; T4: eGF_cys-cr_R ≥90 mL/min/1.73 m². eGFR, estimated glomerular filtration rate; eGFR_cys-cr_, eGFR calculated using a combined creatinine-cystatin C method; eGFR_cys_, eGFR calculated using cystatin C only; eGFR_cr_, eGFR calculated using creatinine only.

Supplementary Table 2. Comparison of long-term adverse prognosis among eGFR Categories.

|  | MACEs | Death | Cardiac death | MI | Unplanned revascularization | Stroke |
| --- | --- | --- | --- | --- | --- | --- |
| eGFR_cys-cr_ < 30 (n=68) | 25 (36.8) | 22 (32.4) | 16 (23.5) | 3 (4.4) | 8 (11.8) | 2 (2.9) |
| 30 ≤ eGFR_cys-cr_ < 60 (n=299) | 37 (12.4) | 28 (9.4) | 17 (5.7) | 9 (3.0) | 26 (8.7) | 12 (4.0) |
| 60 ≤ eGFR_cys-cr_ < 90 (n=661) | 63 (9.5) | 40 (6.1) | 23 (3.5) | 27 (4.1) | 60 (9.1) | 26 (3.9) |
| eGFR_cys-cr_ ≥ 90 (n=372) | 10 (2.7) | 9 (2.4) | 4 (1.1) | 2 (0.5) | 29 (7.8) | 9 (2.4) |
| P value | <0.001 | <0.001 | <0.001 | 0.011 | 0.734 | 0.583 |
| eGFR_cys_ < 30 (n=117) | 40 (34.2) | 34 (29.1) | 24 (20.5) | 6 (5.1) | 15 (12.8) | 5 (4.3) |
| 30 ≤ eGFR_cys_ < 60 (n=545) | 57 (10.5) | 38 (7.0) | 21 (3.9) | 22 (4.0) | 51 (9.4) | 20 (3.7) |
| 60 ≤ eGFR_cys_ < 90 (n=558) | 34 (6.1) | 24 (4.3) | 14 (2.5) | 12 (2.2) | 42 (7.5) | 19 (3.4) |
| eGFR_cys_ ≥ 90 (n=180) | 4 (2.2) | 3 (1.7) | 1 (0.6) | 1 (0.6) | 15 (8.3) | 5 (2.8) |
| P value | <0.001 | <0.001 | <0.001 | 0.280 | 0.290 | 0.909 |
| eGFR_cr_ < 30 (n=41) | 16 (39.0) | 14 (34.1) | 12 (29.3) | 2 (4.9) | 5 (12.2) | 1 (2.4) |
| 30 ≤ eGFR_cr_ < 60 (n=159) | 28 (17.6) | 22 (13.8) | 12 (7.5) | 6 (3.8) | 19 (11.9) | 6 (3.8) |
| 60 ≤ eGFR_cr_ < 90 (n=495) | 50 (10.1) | 35 (7.1) | 23 (4.6) | 18 (3.6) | 36 (7.3) | 21 (4.2) |
| eGFR_cr_ ≥ 90 (n=705) | 41 (5.8) | 28 (4.0) | 13 (1.8) | 15 (2.1) | 63 (8.9) | 21 (3.0) |
| P value | <0.001 | <0.001 | <0.001 | 0.333 | 0.260 | 0.671 |

This table presents the incidence of various adverse cardiovascular events across different eGFR categories as estimated by three different equations. The eGFR categories are defined as follows: T1 (eGFR < 30 mL/min/1.73m²), T2 (30 ≤ eGFR ＜ 60 mL/min/1.73m²), T3 (60 ≤ eGFR ＜ 90 mL/min/1.73m²), and T4 (eGFR ≥ 90 mL/min/1.73m²). MI, myocardial infarction.

Supplementary Table 3. Univariate Cox regression analysis for MACEs.

| Variables | Univariate analysis | | |
| --- | --- | --- | --- |
|  | HR | 95%CI | P |
| Age | 1.073 | 1.054 - 1.092 | <0.001 |
| Female | 1.411 | 0.994 - 2.005 | 0.054 |
| BMI | 0.925 | 0.873 - 0.981 | 0.009 |
| AMI | 1.591 | 1.126 - 2.248 | 0.008 |
| Smoking | 0.752 | 0.536 - 1.055 | 0.099 |
| Previous PCI | 1.389 | 0.824 - 2.342 | 0.217 |
| Hypertension | 1.320 | 0.901 - 1.934 | 0.154 |
| DM | 1.616 | 1.153 - 2.265 | 0.005 |
| SBP | 0.999 | 0.992 - 1.008 | 0.961 |
| Heart rate | 1.016 | 1.005 - 1.028 | 0.004 |
| BNP | 1.001 | 1.000 - 1.001 | <0.001 |
| cTnT | 1.000 | 1.000 - 1.001 | 0.005 |
| Serum creatinine | 1.182 | 1.100 - 1.270 | <0.001 |
| eGFR_cr_ | 1.288 | 1.215 -1.366 | <0.001 |
| eGFR_cys_ | 1.411 | 1.307 - 1.523 | <0.001 |
| eGFR_cys-cr_ | 1.344 | 1.260 - 1.434 | <0.001 |
| FBG | 1.027 | 0.979 - 1.078 | 0.281 |
| TC | 0.978 | 0.854 - 1.119 | 0.742 |
| TG | 0.857 | 0.724 - 1.015 | 0.073 |
| HDL-C | 0.993 | 0.562 - 1.755 | 0.980 |
| LDL-C | 0.969 | 0.805 - 1.166 | 0.739 |
| bSS | 1.054 | 1.037 - 1.070 | <0.001 |
| LVEF | 0.959 | 0.943 - 0.975 | <0.001 |
| β-blockers | 0.967 | 0.672 - 1.393 | 0.858 |
| Diuretics | 3.195 | 2.256 - 4.524 | <0.001 |
| ACEI/ARB | 1.239 | 0.884 - 1.737 | 0.213 |
| Insulin | 1.909 | 1.228 - 2.969 | 0.004 |
| GRACE score | 1.027 | 1.021 - 1.032 | <0.001 |

Hazard ratio (HR) indicates an increased risk for each 10-unit decrease in eGFR. CI, confidence interval; eGFR, estimated glomerular filtration rate; MACEs, Major adverse cardiovascular events; BMI, body mass index; DM, diabetes mellitus; HTN, hypertension; SBP, systolic blood pressure; LVEF, left ventricular ejection fraction; AMI, acute myocardial infarction; bSS, baseline SYNTAX score; cTnT, cardiac troponin T; BNP, brain natriuretic peptide; FBG, fasting blood glucose; TG, triglyceride; TC, total cholesterol; HDL-C, high density lipoprotein cholesterol; LDL-C, low density lipoprotein cholesterol; AMI, acute myocardial infarction; ACEI/ARB, angiotensin converting enzyme inhibitor/angiotensin receptor blocker.

Supplementary Table 4. The comparison of model performance

| variable | δAUC | 95% CI | z | p value |
| --- | --- | --- | --- | --- |
| eGFR_cys-cr_ vs eGFR_cr_ | 0.016 | -0.005 - 0.038 | 1.464 | 0.143 |
| eGFR_cys_ vs eGFR_cr_ | 0.022 | -0.008 - 0.051 | 1.450 | 0.147 |
| eGFR_cys-cr_ vs Scr | 0.088 | 0.054 - 0.122 | 5.045 | < 0.001 |
| eGFR_cys_ vs Scr | 0.093 | 0.053 - 0.134 | 4.547 | < 0.001 |
| eGFR_cr_ vs Scr | 0.072 | 0.042 - 0.102 | 4.717 | ＜0.01 |

δAUC, delta-AUC, which indicates the change in area under the curve; AUC, area under curve; CI, confidence interval; Scr, serum creatinine; MACEs, major adverse cardiovascular events. eGFR_cys-cr_, eGFR calculated using a combined creatinine-cystatin C method; eGFR_cys_, eGFR calculated using cystatin C only; eGFR_cr_, eGFR calculated using creatinine only.

**Supplementary Table 5. The ROC curve analysis of the eGFR_cys-cr_, eGFR_cys_, eGFR_cr_, Scr and Cys-C for MACEs**

|  | AUC | 95% CI | specificity | sensitivity | P value |
| --- | --- | --- | --- | --- | --- |
| **eGFR_cys-cr_** | 0.700 | 0.653 - 0.746 | 58.2 | 71.9 | <0.001 |
| **eGFR_cr_** | 0.683 | 0.637 - 0.730 | 45.0 | 80.7 | <0.001 |
| **eGFR_cys_** | 0.705 | 0.658 - 0.752 | 74.5 | 56.3 | <0.001 |
| **Cys-C** | 0.690 | 0.640 - 0.739 | 72.9 | 55.6 | <0.001 |
| **Scr** | 0.612 | 0.555 - 0.669 | 76.0 | 48.9 | <0.001 |

ROC, receiver operating characteristic; MACEs, major adverse cardiac events; AUC, area under curve; CI, confidence interval; Scr, serum creatinine; Cys-C, Cystatin C; eGFR_cys-cr_, eGFR calculated using a combined creatinine-cystatin C method; eGFR_cys_, eGFR calculated using cystatin C only; eGFR_cr_, eGFR calculated using creatinine only.

Supplementary Table 6. The model performance estimated by internal bootstrap validation method

|  | C-index (95% CI) | P value | Bias-corrected  C-index (95% CI) | P value | Brier score |
| --- | --- | --- | --- | --- | --- |
| Established risk model | 0.770(0.729, 0.811) | ＜0.01 | 0.751(0.711, 0.796) | ＜0.01 | 0.157 |
| Established risk model + eGFR_cr_ | 0.775(0.735, 0.815) | ＜0.01 | 0.753(0.724, 0.800) | ＜0.01 | 0.155 |
| Established risk model + eGFR_cys_ | 0.780(0.739,0.821) | ＜0.01 | 0.760(0.724,0.802) | ＜0.01 | 0.154 |
| Established risk model + eGFR_cys-cr_ | 0.778(0.737,0.819) | ＜0.01 | 0.758(0.727,0.802) | ＜0.01 | 0.154 |

Established risk model was adjusted for age, sex, BMI, HTN, DM, smoking, Heart rate, AMI, LVEF, Diuretics, Insulin, bSS;

HTN, hypertension; DM, diabetes mellitus; eGFR_cys-cr_, eGFR calculated using a combined creatinine-cystatin C method; eGFR_cys_, eGFR calculated using cystatin C only; eGFR_cr_, eGFR calculated using creatinine only; CI, confidence interval.

Supplementary Fig. 1. Calibration plots for adjusted predictive models utilizing eGFR_cys-cr_, eGFR_cys_, and eGFR_cr_. Panels A-C show calibration between predicted and observed probabilities for models predicting MACE. The x-axis represents the predicted adverse cardiovascular events risk. The y-axis represents the actual adverse cardiovascular events rate. The grey line indicates a perfect prediction by an ideal model. The red solid line indicates the performance of the predicting model, of which a closer fit to the grey line suggests better prediction. The adjusted model refers to the established risk model. Established risk model was adjusted for age, sex, BMI, HTN, DM, smoking, Heart rate, AMI, LVEF, Diuretics, Insulin, bSS;


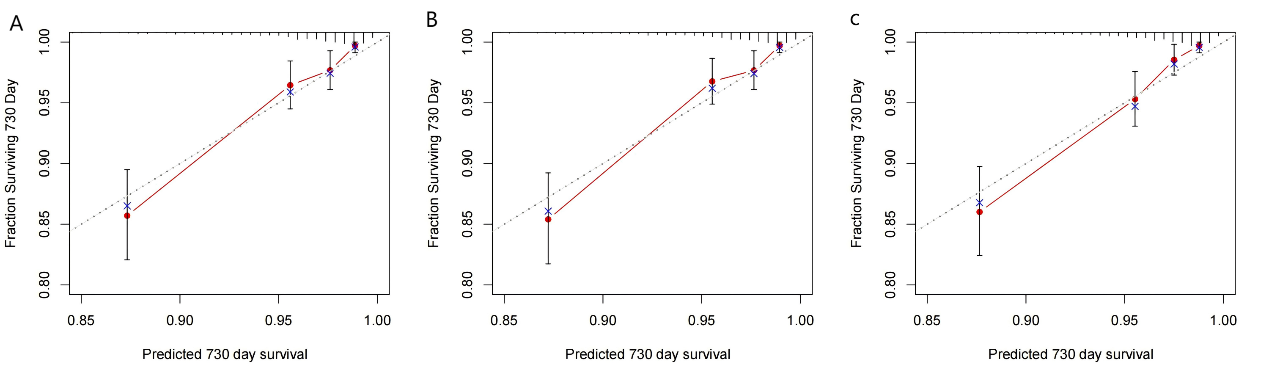

Supplement: Supplementary file 1 [file 2153-8174-26-10-39246-s1.docx]
